# Editorial: Community series - characterization of mobile genetic elements associated with acquired resistance mechanisms, volume II

**DOI:** 10.3389/fmicb.2023.1230730

**Published:** 2023-06-22

**Authors:** John Osei Sekyere, Anusak Kerdsin, Peechanika Chopjitt, Carolin Charlotte Wendling

**Affiliations:** ^1^Medical Diagnostic Laboratories, Genesis Biotechnology Group, Institute of Biomarker Research and Department of Clinical Development, Hamilton Township, NJ, United States; ^2^Department of Dermatology, School of Medicine, Faculty of Health Sciences, University of Pretoria, Pretoria, South Africa; ^3^Faculty of Public Health, Kasetsart University Chalermphrakiat Sakon Nakhon Province Campus, Sakon Nakhon, Thailand; ^4^ETH Zürich, Institute of Integrative Biology, Zurich, Switzerland

**Keywords:** plasmid, integron, transposon, integrative and conjugative element (ICE), mobile genetic element (MGE), antibiotic resistance gene (ARGs), mobile integrative and conjugative elements (MICEs)

Antibiotic resistance in bacteria remains a great challenge to clinical medicine as resistant bacterial infections are very difficult to manage. It is estimated that antibiotic-resistant infections resulted in 1.27 million deaths in 2019, which is expected to increase to 10 million deaths annually by 2050 (Antimicrobial Resistance Collaborators, 2022). In the US alone, at least 2 million people got an antimicrobial-resistant infection, of which at least 23,000 people died in 2019 (CDC, 2019). In the EU, 541,000 deaths were associated with antibiotic resistance while 133,000 deaths were attributable to this menace (European Antimicrobial Resistance Collaborators, 2022). Moreover, the costs associated with antibiotic resistance have been estimated by Nelson et al. (2022) to be $1.9 billion in just a retrospective study. In another study conducted by the CDC and the University of Utah School of Medicine, it was concluded that $4.6 billion in health care costs accrued annually from treating antibiotic resistance in six pathogens in the US (CDC, 2021). These statistics evince why the WHO has categorized antibiotic resistance among the top 10 threats for global health (Antimicrobial Resistance Collaborators, 2022).

As dire as these statistics are, they do not show why antibiotic resistance is spreading so fast among hospital pathogens. The underlying factors driving the acquisition of antibiotic resistance among pathogens are the focus of this Research Topic: mobile genetic elements (MGEs) (Partridge et al., 2018). The acquisition of antibiotic resistance genes (ARGs) via horizontal transfer remains the commonest means of antibiotic resistance transmission. This is a process that can immediately turn a susceptible bacterial strain into a resistant one. Involved in this transmission process are MGEs, which are genetic structures that shuttle resistance genes from chromosome to plasmids, plasmids to plasmids, and bacterial cell to bacterial cell. They include plasmids, transposons, insertion sequences, integrons, ICE (integrative conjugative elements), mobile ICE (MICE), and prophages (Partridge et al., 2018).

The importance of these MGEs to antibiotic resistance transmission was confirmed by da Silva et al. in this series when they undertook a thorough analysis of 345 *Pasteurellaceae* species' genomes (da Silva et al.). In this in-depth analysis, they found that 77.6% of the mobilome (10,820 insertion sequences, 2,939 prophages, and 43 integrative and conjugative elements) integrated into the *Pasteurellaceae* genomes were associated with 55 different ARGs. Evidently, 77.6% of MGEs being associated with the resistome of *Pasteurellaceae* genomes is a substantial number and shows the importance of horizontal gene transfer in antibiotic resistance (da Silva et al.).

Similarly, genomic analysis of the mobilome and resistome of *Streptococcus suis* in pigs found at least 20 integrative and conjugative elements (ICEs) and 10 prophages that were associated with tetracycline, macrolides-Lincosamides-Streptogramins (MLS), and aminoglycoside ARGs (Wang et al.). The ICEs consisted of integrative mobilizable elements (IMEs), *cis-*IMEs (CIMEs), and transposon *Tn*916. These three different ICEs were associated with specific ARGs, underlining their importance in the transmission of clinically important ARGs among pigs and possibly, among human consumers.

*Enterobacterales* are an order of bacteria that consist of important nosocomial pathogens such as *Escherichia coli, Klebsiella pneumoniae*, and *Enterobacter* sp. In a retrospective study spanning between 2009 and 2020, Zelendova et al. identified plasmid-borne *mcr-1, mcr-4*, and *mcr-9* in human clinical *Enterobacterales* isolates in the Czech Republic (Zelendova et al.). Some of these multidrug-resistant isolates also co-harbored carbapenemases such as *bla*_KPC_ and *bla*_OXA − 48_ on plasmids (Kopotsa et al., 2019), making them highly resistant pathogens. Furthermore, three *Enterobacter kobei* isolates co-harbored both *mcr-4* and *mcr-9* ARGs (Zelendova et al.). Instructively, these *mcr* genes were associated with specific plasmid types: *mcr-1* (IncX4, IncH12, and IncI2), *mcr-4* (ColE10), and *mcr-9* (IncH12) (Mmatli et al., 2022).

The importance of IncX-type plasmids in shuttling ARGs, specifically *mcr-1*, was corroborated in Egypt when IncX4 and IncP plasmids were identified to host *mcr-1* in multidrug-resistant *E. coli* strains (Soliman et al.). In these plasmids, the *mcr-1* was sandwiched between two IS*Apl1* insertion sequences to form a composite transposon. A virulence plasmid, hosting important virulence genes, was also detected (Mmatli et al., 2022). These confirm the global distribution of IncX-type plasmids (Kopotsa et al., 2020) and their centrality in spreading ARGs among Enterobacteriaceae, as observed by Guo et al.

This Research Topic thus shows the importance of MGEs in the movement of specific ARGs among bacterial pathogens of the same or different clones and species in humans, animals, and the environment, as well as across borders (Ramaloko and Osei Sekyere, 2022).

## Author contributions

JOS wrote, formatted, and edited the article. All other authors reviewed it. All authors contributed to the article and approved the submitted version.
